# Effect of duration of postherpetic neuralgia on efficacy analyses in a multicenter, randomized, controlled study of NGX-4010, an 8% capsaicin patch evaluated for the treatment of postherpetic neuralgia

**DOI:** 10.1186/1471-2377-10-92

**Published:** 2010-10-11

**Authors:** Lynn R Webster, Marvin Tark, Richard Rauck, Jeffrey K Tobias, Geertrui F Vanhove

**Affiliations:** 1 Lifetree Clinical Research and Pain Clinic, Salt Lake City, Utah, USA; 2 Georgia Medical Research Institute, Marietta, GA, USA; 3 The Center for Clinical Research, Winston-Salem, North Carolina, USA; 4 NeurogesX, Inc., San Mateo, California, USA

## Abstract

**Background:**

Postherpetic neuralgia (PHN) is a painful and difficult to treat complication of acute herpes zoster. Current treatment options provide only partial relief and are often limited by poor tolerability. We evaluated the safety and efficacy of a single 60-minute application of NGX-4010, an 8% capsaicin patch, in patients with PHN.

**Methods:**

This multicenter, double-blind, controlled study randomized 155 patients 2:1 to receive either NGX-4010 or a 0.04% capsaicin control patch. Patients were at least 18 years old with PHN for at least 3 months, and an average Numeric Pain Rating Scale (NPRS) score of 3 to 9. The primary efficacy endpoint was the percentage change in NPRS score from baseline to weeks 2-8.

**Results:**

The mean percent reduction in "average pain for the past 24 hours" NPRS scores from baseline to weeks 2-8 was greater in the NGX-4010 group (36.5%) compared with control (29.9%) although the difference was not significant (p = 0.296). PGIC analysis demonstrated that more NGX-4010 recipients considered themselves improved (much, or very much) compared with control at weeks 8 and 12, but the differences did not reach statistical significance. Post hoc analyses of patients with PHN for at least 6 months showed significantly greater reductions in "average pain for the past 24 hours" NPRS scores from baseline to weeks 2-8 in NGX-4010 patients compared to controls (37.6% versus 23.4%; p = 0.0291). PGIC analysis in this subgroup demonstrated that significantly more NGX-4010 recipients considered themselves much or very much improved compared with control at week 12 (40% versus 20%; p = 0.0403;).

**Conclusions:**

Although treatment appeared to be safe and well tolerated, a single 60-minute application of NGX-4010 failed to show efficacy in this study which included patients with PHN for less than 6 months. Large reductions in pain observed among control patients with pain for less than 6 months may have been due to spontaneous resolution of PHN, may have confounded the results of the prespecified analyses, and should be taken into account when designing PHN studies.

**Trial Registration:**

NCT00068081

## Background

Postherpetic neuralgia (PHN) is a painful complication of acute herpes zoster (shingles) that is caused by reactivation of the varicella zoster virus usually contracted in childhood. Acute herpes zoster is often very painful. Usually this pain subsides, but if it persists beyond healing, it is referred to as PHN. Transition from acute herpes zoster to PHN occurs when the pain in the affected area persists months after crusting of the skin lesions. Definitions of PHN vary from as short as 1 month to as long as 6 months after lesion crusting [1]. The prevalence of PHN increases with age with between 25% and 50% of adults older than 50 years, and up to 75% of patients with herpes zoster over 70 years, developing PHN [2-4].

The treatment of peripheral neuropathic pain syndromes commonly requires the use of more than one neuropathic pain medication [5-7] such as anticonvulsants (pregabalin and gabapentin), topical lidocaine, opioids, tricyclic antidepressants, and selective serotonin and norepinephrine reuptake inhibitors (SSNRIs). Despite the availability of various options, most of these treatments provide only partial relief [8-11] and their use can be limited by poor tolerability issues such as central nervous system side effects, the need for titration, and administration of multiple daily doses.

Sensitization of peripheral nociceptors that express transient receptor potential vanilloid 1 receptor (TRPV1), a ligand-gated non-selective cation channel, is thought to play a role in PHN [1,12]. Treatment strategies directly targeting TRPV1 have been developed. Capsaicin is a highly selective activating ligand for TRPV1, and causes depolarization, action potential initiation, and the transmission of pain signals resulting in a burning sensation, hyperalgesia, allodynia, and erythema. Following continued capsaicin exposure, however, TRPV1-containing sensory axons become defunctionalized, preventing pain transmission and resulting in a reduced pain response [13,14]. Low-concentration capsaicin creams (0.025% and 0.075%) have demonstrated efficacy in the treatment of PHN [4,15,16]. However, they require multiple daily applications over several weeks to achieve significant pain relief and cause a burning sensation at the application site both of which may lead to a lack of compliance. NGX-4010 is a high-concentration capsaicin dermal patch (capsaicin, 8%) developed to rapidly deliver a therapeutic dose of capsaicin locally into the skin. A single 60-minute application of NGX-4010 has been shown to reduce pain in controlled clinical trials in patients with PHN [17-19]. We report the results of a multicenter, double-blind, controlled, phase 3 study that evaluated the safety and efficacy of a single application of NGX-4010 in patients with PHN. In contrast to the other NGX-4010 studies that only allowed PHN patients to enroll if at least 6 months had elapsed since vesicle crusting, patients in this study were allowed to enroll if at least 3 months had elapsed since vesicle crusting

## Methods

### Patients

Patients 18 years or older with a diagnosis of PHN and an average Numeric Pain Rating Scale (NPRS) [9] score of 3 to 9 (inclusive) were eligible if at least 3 months had elapsed since vesicle crusting. Patients taking chronic pain medications had to be on a stable dose of those medications for at least 21 days before the day of study patch application and remain on a stable dose throughout the study period. It was prespecified that at least 25% of patients would not be taking concomitant pain medications at entry. Women of childbearing age were required to have a negative pregnancy test and be willing to use an effective method of contraception for 30 days after exposure to study medication.

Exclusion criteria were as follows: use of any topically applied pain medication on the painful area within 21 days before the day of application of the study patch; current use of any investigational drug or class 1 anti-arrhythmic drug; uncontrolled diabetes mellitus or uncontrolled hypertension; significant pain of an etiology other than PHN; painful PHN areas located only on the face, above the scalp hairline, or near mucous membranes; and hypersensitivity to capsaicin, local anesthetics, oxycodone hydrochloride, hydrocodone, or adhesives. As prior use of high-dose opioids could limit the responsiveness to the optional oral opioid rescue analgesics used during the treatment procedure, patients using concomitant opioid medication that were not orally or transdermally administered or exceeded a total dose of 60 mg/day morphine equivalent were excluded.

The study was approved by Institutional Review Boards at all participating sites, and conducted in accordance with the ethical principles of the Declaration of Helsinki, Good Clinical Practice guidelines, and applicable regulatory requirements. Written informed consent was obtained from all participating patients before initiating study-related procedures.

### Procedures

A baseline screening period was followed by a treatment day (day 0), a 12-week post-treatment assessment period with clinic visits at weeks 4, 8, and 12. Eligible patients were randomized 2:1 to receive either NGX-4010 (capsaicin 640 μg/cm^2^, 8%, NeurogesX Inc., San Mateo, CA) or an identically appearing low-concentration capsaicin control patch (capsaicin 3.2 μg/cm^2^, 0.04%) for 60 minutes according to a randomization scheme prepared by Cardinal Health (Morrisville, NC). The low-concentration capsaicin control patches were used in place of placebo patches to provide effective blinding in the study since topical capsaicin can produce local erythema and a burning sensation. All patients were pre-treated with a topical anesthetic cream (ELA-Max4^®^, lidocaine 4%; Ferndale Laboratories, Inc., Ferndale, MI) for 60 minutes before the application of the study or control patch(es) which were applied directly to the painful area(s) (up to 1000 cm^2^). After patch removal, the area was cleansed with a proprietary cleansing gel formulated to remove residual capsaicin. Patients were monitored for 2 hours after patch removal. Local cooling as well as oxycodone hydrochloride oral solution (1 mg/mL) or equivalent could be administered at the onset of treatment-associated discomfort and as needed. Patients could take opioid rescue medication (hydrocodone bitartrate/acetaminophen 5 mg/500 mg) every 8 hours for up to 3 days after patch application for treatment-associated discomfort as needed. Topical pain medications were not permitted during the 12-week study period. Patients were allowed to take acetaminophen up to 2 g/day as needed for aches and pains.

### Efficacy Measures and Data Analysis

Efficacy was evaluated with daily Numeric Pain Rating Scale (NPRS) scores throughout the 12-week study period. The NPRS is an 11-point scale (0 to 10) with 0 indicating no pain and 10 indicating the worst possible pain [20]. Patients recorded NPRS scores for "worst pain for the past 24 hours", "average pain for the past 24 hours", and "pain now" in a take home diary beginning on the evening of the Treatment Visit (Day 0) through the evening before the week 12 visit. Patient Global Impression of Change (PGIC; patients reported how they felt compared to baseline on a scale of -3 indicating "very much worse" to +3 indicating "very much improved" with 0 being "no change") and investigator-rated Clinical Global Impression of Change (CGIC) [21] were evaluated at weeks 4, 8 and 12. The modified Brief Pain Inventory (BPI) [22] was collected at screening and at weeks 4, 8 and 12. The Short-Form McGill Pain Questionnaire (SFMPQ) [23] was collected at screening and at weeks 4, 8 and 12.

The primary efficacy endpoint was the percentage change in "average pain for the past 24 hours" NPRS scores from baseline to weeks 2 through 8. To avoid the potential confounding effect of allowed opioid rescue medications during days 0 to 3, week 1 NPRS scores were not included in the primary analysis. Other efficacy measures included: percentage change in NPRS scores from baseline to weeks 2-4 and 2-12; the percentage of patients with a ≥ 30% and ≥ 50% reduction in NPRS score from baseline to weeks 2-4, 2-8 and 2-12; the percentage of patients considered improved (much, or very much) on the PGIC and CGIC at weeks 4, 8 and 12; changes from screening in the BPI questionnaire collected at weeks 4, 8 and 12; and changes from screening in the SFMPQ questionnaire collected at weeks 4, 8 and 12. Weekly changes in NPRS scores were also performed.

Efficacy analyses were based on the intent-to-treat population that consisted of all patients who received any study treatment and had at least 3 days of available NPRS scores during the baseline period. The NGX-4010 group was compared to the control group using a gender-stratified ANCOVA model with baseline pain as the only covariate. The same method was used to analyze the differences between the NGX-4010 groups and the control group in NPRS scores for weeks 2-4 and 2-12. Logistic regression with gender and baseline as covariates tested the difference in the proportion of patients with a ≥30% and a ≥50% mean decrease from baseline in NPRS scores during weeks 2-4, 2-8 and 2-12. For the Short-Form McGill Pain Questionnaire and BPI, a t-test was used to test for differences in change from screening to week 8 between treatment groups. For PGIC and CGIC, Fisher's exact test was used to test for differences between treatment groups in the percentage of patients considered improved (much, or very much). To assess the potential impact of enrolling patients with disease of less than 6 months duration, post hoc analyses were performed on the subgroup of patients with PHN duration of at least 6 months (180 days). Treatment comparisons were performed using gender-stratified ANCOVA to test for a difference between the NGX-4010 and control groups, with baseline pain, pre-anesthetic pain score, and percent change in NPRS score from pre-anesthetic to pre-patch application time point as covariates. The percent change in pain following application of the topical anesthetic and the pain score reported immediately before application of the topical anesthetic were found to be significant covariates in these post hoc analyses and included in the model.

Missing post-treatment NPRS scores were imputed using a modified last-observation-carried-forward approach. If the NPRS score was missing on days 0-8, the baseline score was imputed for that day. If the NPRS score was missing for any day past day 8, then the latest available non-missing score collected before that day was imputed for that missing value. If NPRS scores were missing for all post treatment study days (including day 0), then the baseline score was imputed for all missing scores. No imputation was used for the calculation of weekly scores. For the calculation of NPRS baseline scores, all available screening scores which were not biased by pain medication changes were used. For changes in non-SSRI antidepressant or anticonvulsant medications, pain scores up to 14 days after the medication change were considered biased. For changes in other pain medications, pain scores up to the day of medication change were considered biased. Changes in minor OTC analgesics (acetaminophen, aspirin) were ignored.

It was estimated that to achieve 90% power at the 0.05 significance level, a total of 150 patients, with 100 designated for NGX-4010 and 50 designated for control treatment, were required to detect a difference of 15% in change from baseline in NPRS scores between the NGX-4010 and control group.

### Safety Measures and Data Analysis

Safety was assessed by continuous monitoring of adverse events and periodic assessments of clinical laboratory parameters, vital signs, physical examinations, dermal assessments (0- to 7-point severity score) [24], pain experienced during and after the patch application by using NPRS scores on the day of treatment (prior to topical local anesthetic application, 5 minutes prior to patch application, 5 minutes prior to patch removal and 1 hour after patch removal), and rescue medication and concomitant medication usage. Treatment associated erythema, discomfort and pain on the day of treatment were not captured as adverse events but reported as dermal assessment scores or "Pain Now" NPRS scores. Standardized neurosensory examinations (allodynia, light brush, pinprick, warmth and vibration) were performed at screening, week 4, week 8 and 12. Changes in neurosensory assessments from screening to each assessment time point were categorized using prespecified algorithms. On the day of treatment, the proportions of patients reporting each level of dermal response, the "Pain Now" NPRS score and the change in "Pain Now" NPRS score from the pre-local anesthetic time point, vital signs (systolic blood pressure, diastolic blood pressure, heart rate, and respiratory rate), change in vital signs from the pre-local anesthetic time point and the number and proportion of patients who had shorter patch application times (< 90% of intended patch application duration) were summarized. The proportion of patients classified as "same or no increase", " < 33% increase", or " > 33% increase" in maximum pain score from baseline during the first 48 hours were also summarized.

Adverse events were coded using the Medical Dictionary for Regulatory Activities (MedDRA; Version 7.0). Rescue medication use from days 0 to 5, the number of patients completing the intended patch duration, demographics, and baseline clinical characteristics were compared between groups with Fisher exact tests or *t-*tests, as appropriate. The proportions of patients reporting each level of maximum dermal response on the day of treatment were compared between the active group and the control group using Fisher's exact test. The results of the allodynia assessments at each visit were compared using a t-test while the rest of the neurologic sensory exams were compared using a Cochran-Mantel-Haenszel test for trend.

## Results

### Patients

**Figure 1:**
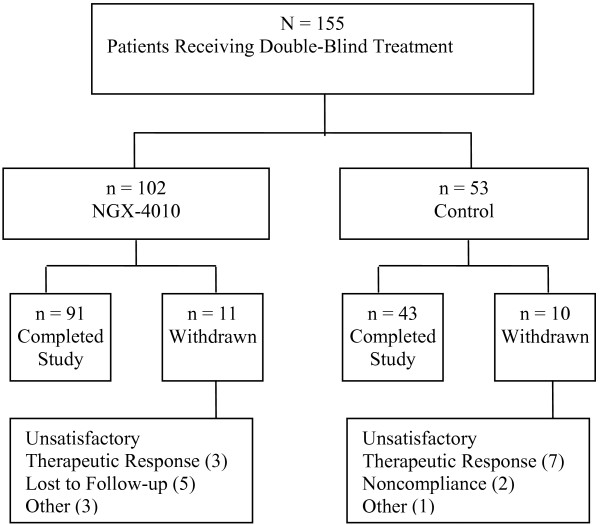
**Study randomization and disposition**.

A total of 155 patients were enrolled into the study and received double-blind treatment: 102 patients received NGX-4010 and 53 patients received control (Figure 1). A total of 134 (86%) patients completed the 12-week study: 91 (89%) patients in the NGX-4010 group and 43 (81%) patients in the control group. The most common reasons for premature termination included unsatisfactory therapeutic response (NGX-4010: n = 3; Control: n = 7) and lost to follow-up (NGX-4010: n = 5; Control: n = 0). No patients terminated the study early due to adverse events and no patients died during the study.

Demographics, baseline pain scores, duration of PHN, the size of the painful area, the size of the treated area, and use of baseline concomitant pain medications (opioids, antidepressants, anticonvulsants and other) were similar between treatment groups (Table 1).

**Table 1:** Patient demographics and clinical characteristics

	NGX-4010 (n = 102)	Control (n = 53)
**Demographics**		

Age (yr), mean ± SD	68.7 ± 12.00	71.2 ± 11.26
Male, n (%)	47 (46%)	25 (47%)
Race		
White	89 (87%)	45 (85%)
Black or African American	6 (6%)	3 (6%)
Asian	1 (1%)	1 (2%)
American Indian or Alaska Native	0 (0%)	1 (2%)
Native Hawaiian or Other Pacific Islander	0 (0%)	0 (0%)
Other	6 (6%)	3 (6%)

**Clinical characteristics**		

Duration of Pain (yr), mean ± SD	2.7 ± 2.92	3.4 ± 4.00
Baseline ^a ^"Average Pain for the Past 24 Hours", mean ± SD	5.4 ± 1.58	5.3 ± 1.53
On Concomitant Pain Medication^b^, n (%)	78 (76%)	42 (79%)
Opioids	26 (25%)	13 (25%)
Anticonvulsants	26 (25%)	18 (34%)
Antidepressants	10 (10%)	6 (11%)
Other	52 (51%)	30 (57%)
Size of Painful Area at Screening (cm^2^), mean ± SD	337.9 ± 226.46	334.2 ± 221.92

Baseline pain level was defined as the mean of all available non-biased Screening NPRS scores in that category.

A patient was defined as being on concomitant pain medication if pain medication started prior to day of treatment, continued on the day of treatment, and was taken for at least 7 days.

### Efficacy

Patients treated with a single 60-minute application of NGX-4010 reported a mean 36.5% reduction in pain during weeks 2 to 8 compared with 29.9% in the control group (p = 0.296; Table 2). Forty-nine percent of NGX-4010 patients were considered to have responded to treatment (i.e., experienced a ≥ 30% mean decrease from baseline in pain) during weeks 2 to 8 compared with 45% of controls (p = 0.574). The proportion of patients who achieved a ≥ 50% decrease in pain scores from baseline to weeks 2 to 8 was similar between the treatment groups (36%). Similar results were observed during weeks 2 to 12. The results of the analyses for change in "worst pain for the past 24 hours" and "pain now" scores from baseline to weeks 2-8 and 2-12 were comparable to the results for change in "average pain for the past 24 hours".

**Table 2:** Clinical efficacy of NGX-4010 in all patients with PHN

NPRS Scores	NGX-4010 (n = 102)	Control (n = 53)	P-value
Baseline, LS mean (SE)	5.4 (0.16)	5.3 (0.22)	
95% CI	5.12, 5.73	4.87, 5.72	
Change, mean (SE)			
Baseline to weeks 2 to 8	-1.8 (0.20)	-1.6 (0.27)	0.462
95% CI	-2.23, -1.45	-2.13, -1.05	
Baseline to weeks 2 to 12	-1.8 (0.20)	-1.7 (0.28)	0.750
95% CI	-2.25, -1.45	-2.29, -1.18	
Percent Change, mean (SE)			
Baseline to weeks 2 to 8	-36.5 (3.68)	-29.9 (5.10)	0.296
95% CI	-43.72, -29.19	-39.94, -19.78	
Baseline to weeks 2 to 12	-36.6 (3.75)	-32.3 (5.21)	0.509
95% CI	-44.02, -29.19	-42.63, -22.05	
Patients with ≥ 30% reduction, %			
Baseline to weeks 2 to 8	49	45	0.574
Baseline to weeks 2 to 12	49	49	0.956
Patients with ≥ 50% reduction, %			
Baseline to weeks 2 to 8	36	36	0.930
Baseline to weeks 2 to 12	39	36	0.628
PGIC			
Improved (very much, much)			
Week 4, n	100	49	
N (%)	40 (40)	19 (39)	1.0000
Week 8, n	91	43	
N (%)	43 (47)	14 (33)	0.1351
Week 12, n	95	50	
N (%)	41 (43)	15 (30)	0.1518

**Figure 2:**
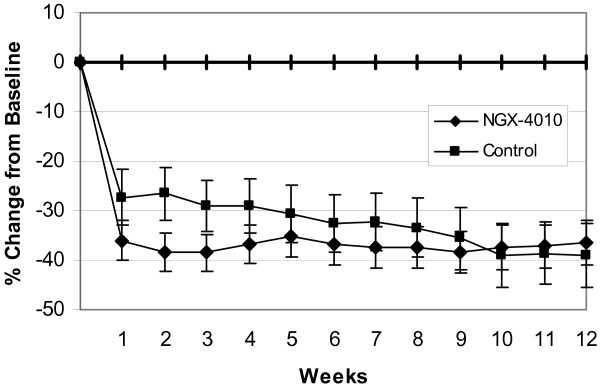
**Percent change from baseline in "average pain for the past 24 Hours" NPRS scores by week**. Values shown are mean and standard error of the mean (SEM). NGX-4010 showed greater improvement in pain scores at weeks 1 through 8 but the differences between the two groups diminished over time largely due to continued improvement in the control group.

During week 1, patients receiving NGX-4010 experienced a 36.0% reduction in 24 hour average pain scores compared to baseline (Figure 2). The observed pain reduction remained relatively constant over the entire 12 week study period. The mean percent change from baseline in 24 hour average pain scores was approximately 5%-10% greater in the NGX-4010 group relative to the control group at weeks 1 through 8. Differences between the groups diminished over time, largely due to continued improvement in the control group.

Although more NGX-4010 recipients considered themselves to have much, or very much improved compared with controls at weeks 8 and 12, the differences did not reach statistical significance (Table 2). The results of the CGIC were comparable to the results of the PGIC. More NGX-4010 patients were judged by the study investigators to have been much or very much improved compared to control patients at week 4 (46% vs. 37%), week 8 (51% vs. 40%) and week 12 (46% vs. 32%) but the differences were not statistically significant. Other patient-rated questionnaires (BPI and SFMPQ) showed no significant differences between the NGX-4010 and control group.

To assess the potential impact of enrolling patients with disease of less than 6 months duration, post hoc analyses were performed on the subgroup of patients with PHN duration of at least 6 months (180 days). Sixteen (16%) patients enrolled in the NGX-4010 group and 10 (18%) patients in the control group had PHN duration between 3 and 6 months and were excluded form these analyses. A summary of the "average pain for the past 24 hours" NPRS scores for weeks 2-8 and 2-12 as well as the results of the responder and PGIC analyses for this subgroup are presented in Table 3. Demographics, baseline pain scores, duration of PHN, the size of the painful area, the size of the treated area, and use of baseline concomitant pain medications were similar between treatment groups in this subset of patients. Patients with PHN for at least 6 months receiving NGX-4010 had a mean percent change from baseline during weeks 2 to 8 of -37.6% compared to -23.4% in controls (p = 0.0291). A greater proportion of NGX-4010 patients (49%) were considered to have responded to treatment (i.e., experienced a ≥ 30% mean decrease from baseline in pain) during weeks 2 to 8 compared with 40% of controls (*P *= 0.090). Results for this subgroup were similar to the results from the subgroup with PHN duration of less than 6 months with regard to patients treated with NGX-4010 who showed a mean percent change from baseline during weeks 2 to 8 of -36.8% (data not shown); however, control patients with PHN duration of 6 months or more had a smaller mean percent change from baseline during weeks 2 to 8 compared to control patients in the subgroup with PHN duration of less than 6 months (-23.4% versus -47.1%, respectively).

**Table 3:** Clinical efficacy of NGX-4010 in patients with PHN duration ≥ 6 months (180 days)

NPRS Scores	NGX-4010 (n = 86)	Control (n = 43)	P-value
Baseline, LS mean (SE)	5.4 (0.17)	5.2 (0.24)	
95% CI	5.05, 5.72	4.77, 5.72	
Change, mean (SE)			
Baseline to weeks 2 to 8	-1.8 (0.20)	-1.4 (0.29)	0.072
95% CI	-2.18, -1.38	-1.92, -0.79	
Baseline to weeks 2 to 12	-1.8 (0.20)	-1.3 (0.28)	0.160
95% CI	-2.23, -1.44	-1.90, -0.78	
Percent Change, mean (SE)			
Baseline to weeks 2 to 8	-37.6 (3.66)	-23.4 (5.21)	0.0291
95% CI	-44.85, -30.35	-33.74, -13.10	
Baseline to weeks 2 to 12	-37.5 (3.76)	-25.5 (5.35)	0.069
95% CI	-44.98, -30.10	-36.06, -14.88	
Patients with ≥ 30% reduction, %			
Baseline to weeks 2 to 8	49	40	0.090
Baseline to weeks 2 to 12	50	44	0.277
Patients with ≥ 50% reduction, %			
Baseline to weeks 2 to 8	35	30	0.270
Baseline to weeks 2 to 12	40	28	0.065
PGIC			
Improved (very much, much)			
Week 4, n	84	40	
N (%)	31 (37)	14 (35)	1.0000
Week 8, n	78	35	
N (%)	34 (44)	10 (29)	0.1487
Week 12, n	81	41	
N (%)	32 (40)	8 (20)	0.0403

Analysis of PGIC in the subgroup of patients with PHN for 6 months or longer demonstrated that significantly more NGX-4010 recipients considered themselves to have much or very much improved compared with the control group at week 12 (40% vs. 20%; p = 0.0403; Table 3). The results of the CGIC were comparable to the results of the PGIC. More NGX-4010 patients were judged by the study investigators to have been much or very much improved compared to control patients at week 4 (42% vs. 35%), week 8 (49% vs. 35%) and week 12 (43% vs. 24%) but the differences were statistically significant only at week 12 (p = 0.0485). Analyses of other patient-rated questionnaires in this subgroup (BPI and SFMPQ) showed no significant differences between the NGX-4010 and control group.

### Safety

**Figure 3:**
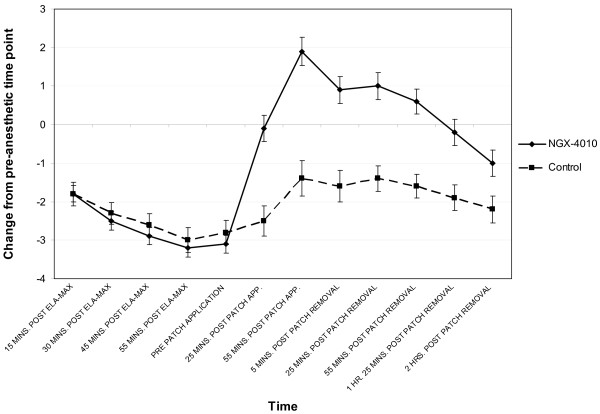
**Change in "Pain Now" NPRS scores from pre-anesthetic time point during the treatment procedure**. Values shown are mean and SEM. "Pain Now" NPRS scores decreased following topical anesthetic and increased following patch application in both treatment groups. Patients receiving NGX-4010 reported a mean maximum increase of 1.9 from the pre-anesthetic time point. NPRS scores did not increase above pre-procedure levels in those receiving control. NPRS scores in the NGX-4010 group decreased to below pre-procedure levels at 85 minutes after patch removal.

NGX-4010 was well tolerated in the majority of patients. All but 4 of 102 patients (96%) in the NGX-4010 group completed at least 90% of the intended patch application duration. Those patients that did not complete the intended patch application duration had the patches removed early due to application site pain or burning. All patients in the control group completed at least 90% of the intended patch application duration. On the day of treatment, mean "Pain Now" NPRS scores decreased following topical anesthetic and increased following patch application in both treatment groups (Figure 3). Patients receiving NGX-4010 reported a mean maximum increase of 1.9 from the pre-anesthetic time point during and after patch application. In contrast, though control patch application resulted in an increase in NPRS scores, NPRS scores did not increase above pre-procedure levels. After patch removal, NPRS scores in the NGX-4010 group decreased to below pre-procedure levels at 85 minutes after patch removal. During the first 48 hours, a ≥ 33% increase from baseline in NPRS score after patch application was reported in 73% of patients treated with NGX-4010 and 51% of controls. On the evening of the day of treatment, all treatment groups had reductions in "pain now" scores compared to baseline though the reductions were smaller in the NGX-4010 group (-1.6, -28.0%) compared to control (-2.3, -34.9%).

Treatment-related pain was manageable in most patients with the use of local cooling or short-acting oral opioids. Thirty-four percent of NGX-4010 patients and 9% of control patients received oxycodone on the day of treatment. The mean dose of oxycodone used was 10.6 ± 5.43 (SD) mg for patients treated with NGX-4010 and 5.6 ± 1.34 (SD) mg for patients treated with control (p = 0.0010). Few patients used hydrocodone/acetaminophen for treatment-related pain from days 0-5: 12% of NGX-4010 patients and 2% of controls.

A total of 76 NGX-4010 patients (75%) and 28 (53%) control patients reported adverse events. The most frequently reported adverse events were related to local application site reactions (pruritus, dryness and swelling). These application site reactions occurred more commonly in NGX-4010 patients (Table 4), were mostly mild or moderate in severity and transient. Treatment associated erythema, discomfort and pain on the day of treatment were not captured as adverse events but reported as dermal assessment scores or NPRS scores. Serious adverse events occurred in 7% and 0% of NGX-4010 and control patients, respectively. These events consisted of atrial fibrillation, cardiac valve disease, myocardial infarction, tachycardia, diarrhea, food poisoning, nausea, vomiting, cholelithiasis, viral gastroenteritis, rib fracture, arthritis, malignant lung neoplasm, pneumothorax and orthostatic hypertension. Only one (myocardial infarction) occurred in more than 1 patient (in two). No serious adverse event was considered related to treatment.

**Table 4:** Most frequently (≥3% of patients) reported adverse events

System Organ Class Preferred Term, n (%)	NGX-4010 (n = 102)	Control (n = 53)
**Number of Patients Reporting 1 or More Adverse Events**	**76 (75%)**	**28 (53%)**

**Gastrointestinal Disorders**	**10 (10%)**	**4 (8%)**
Nausea	5 (5%)	1 (2%)
**General Disorders and Administration Site Conditions**	**38 (37%)**	**12 (23%)**
Application Site Burning	3 (3%)	0
Application Site Discoloration	0	3 (6%)
Application Site Dryness	10 (10%)	2 (4%)
Application Site Erythema	4 (4%)	0
Application Site Pain	4 (4%)	2 (4%)
Application Site Papules	4 (4%)	2 (4%)
Application Site Pruritus	17 (17%)	6 (11%)
Application Site Swelling	10 (10%)	1 (2%)
Application Site Urticaria	3 (3%)	0
Application Site Vesicles	5 (5%)	1 (2%)
Pain Exacerbated	3 (3%)	0
**Infections and Infestations**	**16 (16%)**	**8 (15%)**
Bronchitis	3 (3%)	0
Herpes Zoster	3 (3%)	2 (4%)
Upper Respiratory Tract Infection	1 (1%)	2 (4%)
**Injury, Poisoning and Procedural Complications**	**4 (4%)**	**6 (11%)**
Injury	1 (1%)	2 (4%)
**Musculoskeletal and Connective Tissue Disorders**	**10 (10%)**	**2 (4%)**
Back Pain	3 (3%)	1 (2%)
**Nervous Systems Disorders**	**5 (5%)**	**5 (9%)**
Dizziness	1 (1%)	3 (6%)
**Respiratory, Thoracic and Mediastinal Disorders**	**12 (12%)**	**4 (8%)**
Cough	3 (3%)	0
Nasal Congestion	1 (1%)	2 (4%)
Nasopharyngitis	3 (3%)	2 (4%)
**Vascular Disorders**	**5 (5%)**	**1 (2%)**
Hypertension	3 (3%)	0

Dermal irritation was generally mild and transient. At patch removal, 83% of NGX-4010 patients and 62% of control patients had dermal scores of two or more (definite erythema or minimal edema or minimal papular response). Two hours after patch removal, 52% of NGX-4010 patients had dermal scores of 2 or more compared to 4% of controls. Few NGX-4010 patients (< 3%) had a dermal assessment above 2 at any time point. By week 4, the majority of patients in the both the NGX-4010 (92%) and control (89%) groups had no evidence of dermal irritation (score = 0) and no patients in either the NGX-4010 or control groups had dermal assessment scores ≥ 2.

There were no statistically significant differences between the treatment groups in light brush, pinprick, vibration, and warmth sensations at screening, week 4, week 8, or week 12. No trends consistent with a decrease in sensory function were observed in either group over the 12-week study period. Though patients treated with NGX-4010 demonstrated a larger reduction in mean surface area of allodynia from screening to week 8 (-43.4 cm^2^) compared to control (-25.1 cm^2^), the results were highly variable and not statistically significantly different.

Small, transient changes in blood pressure were noted during and shortly after the treatment procedure. Blood pressure decreased following topical anesthetic application and increased after patch application in the NGX-4010 group, while blood pressure remained at or below the pre-treatment level during patch treatment through 2 hours post-treatment in the control group. These changes paralleled increases in pain as measured by NPRS scores during this period. For the NGX-4010 group, mean increases were ≤ 7.9 mm Hg in systolic blood pressure and ≤ 3.3 mm Hg in diastolic blood pressure. Blood pressure began returning toward pretreatment values within 60 minutes after patch removal. One subject treated with NGX-4010 experienced an adverse event of increased blood pressure on the day of treatment that was not considered related to treatment.

There was no evidence of an effect of NGX-4010 on any laboratory parameter evaluated. Hematologic and serum chemistry laboratory values showed no trends for any parameter during the 12-week study in either treatment group. No other safety concerns were identified.

## Discussion

This randomized, double-blind, controlled study in patients with PHN for at least three months shows that following treatment with NGX-4010, pain scores began declining as early as the evening of the day of treatment and were reduced by approximately 36% during week 1. The pain reduction remained relatively constant over the over the entire 12 week study period. While pain reduction in the NGX-4010 group was 5-10% higher than the control group during the first 8 weeks, differences between the groups diminished over time; this was largely due to continued improvement in the control group. Spontaneous improvement of PHN is often observed, primarily during the first 6 months after onset of the disease [25]. Since this study required patients with only 3 months after vesicle crusting and other studies of NGX-4010 conducted in PHN required 6 months post vesicle crusting [17-19], exploratory post hoc analyses of patients with PHN for 6 months or more, a population that would be less likely to experience spontaneous improvement, were also performed. A significantly greater reduction in pain was observed in patients treated with NGX-4010 compared to control for weeks 2-8 in this subgroup. The response in control patients with PHN for 6 months or more was lower than that observed in the cohort with PHN for less than 6 months while the response in patients treated with NGX-4010 was similar to that observed in the cohort with PHN for less than 6 months. Given these findings, it is possible that spontaneous improvement in those patients with PHN of less than 6 months duration confounded the overall study results. Studies of NGX-4010 that required patients to have PHN for at least 6 months have demonstrated that a single application of NGX-4010 can provide pain relief that can be maintained for up to 12 weeks following treatment [17-19]. Overall, these findings suggest that, to reduce the potential confounding effect of spontaneous resolution, PHN studies should consider limiting enrollment to patients with PHN for at least 6 months duration.

Another possible factor confounding the results of this study is the use of a low-concentration capsaicin control patch instead of an inert placebo. A patch containing a low-concentration of capsaicin was chosen as a control to address the difficulty in blinding the use of topical capsaicin. The low-concentration patch delivered an amount of capsaicin that, like NGX-4010, was capable of producing local application site reactions. Erythema, pain, and rescue medication usage occurred with both capsaicin formulations suggesting that the blind could not be broken based on an individual patient's initial reaction to treatment. The use of a low-concentration capsaicin control patch may have led to an underestimation of the efficacy of NGX-4010 either because of a possible intrinsic analgesic effect of the low-concentration capsaicin control patch or because of an enhanced placebo response due to application site reactions that resulted from the capsaicin in the control.

Lastly, power estimations for this study were calculated using a 15% difference between the treatment groups. This estimate was based on the results of the first randomized, controlled NGX-4010 study in PHN patients which showed a small pain reduction in the control arm (-4.4%) and a 28% difference in NPRS score reduction between NGX-4010 and control during weeks 2-4 [17]. Subsequent studies, however, have shown much larger reductions in the control arms and treatment differences that were less than those observed in the first study [18,19]. Therefore, the sample size in this study was not large enough to show a significant difference between the two treatment groups.

Treatment with NGX-4010 was well tolerated in the majority of patients, with nearly all patients completing the full duration of treatment. Although an initial increase in pain was evident during and after NGX-4010 patch application, pain returned to near pre-procedure levels within 85 minutes after patch removal and by the evening of the day of treatment, patients had on average less pain compared with baseline. Mild, transient dermal irritation was observed in a majority of patients after patch removal. Capsaicin-related local application site reactions were the most common adverse events and were transient, mostly mild to moderate, and self-limited. Application site pain could be adequately managed by pre-treatment with a local anesthetic and local cooling or, if needed, short-acting oral opioid analgesics. Small transient increases in systolic and diastolic blood pressure seen during or shortly after treatment were likely due to treatment-associated discomfort.

No reduction in sensory function following NGX-4010 administration was observed suggesting that capsaicin treatment can lead to pain reduction without clinically relevant changes in protective sensation. Indeed, when used as a topical analgesic, capsaicin's mechanism of action involves the selective and reversible defunctionalization of cutaneous sensory nerve endings expressing TRPV1, which have been shown to be hyperactive in painful peripheral neuropathies. The high selectivity of capsaicin for the TRPV1 receptor, coupled with the selective expression of TRPV1 in nociceptive sensory nerves, suggests that even with defunctionalization of cutaneous nociceptors, other skin sensory nerve endings that are capsaicin-insensitive, including those that arise from Aβ-fibers that transduce tactile and proprioceptive stimuli, will be intact and functional [13,26]. In addition, only a subpopulation of Aδ-fibers expresses TRPV1 and at least 6 other TRP channels (TRPV2, TRPV3, TRPV4, TRPM2, TRPM4, and TRPM5) participate in hot/warm thermal perception [26,27].

## Conclusion

Although treatment appeared to be safe and well tolerated, a single application of NGX-4010 failed to show efficacy in this study which included patients with PHN for less than 6 months. Spontaneous resolution of PHN, which is primarily observed during the first 6 months, may have confounded the results. Therefore, when designing PHN studies, the potential for spontaneous resolution should be taken into account.

## Competing interests

LRW, MT and RR were compensated by NeurogesX for their roles as principal investigators. LRW and RR are consultants for Neurogesx and Astellas. JKT and GFV are employees of NeurogesX and own NeurogesX stock.

## Authors' contributions

LRW, MT and RR were principal investigators in the study and made substantial contributions to acquisition of data and drafting of the manuscript. JKT and GFV interpreted the results and drafted the manuscript. All authors read and approved the final manuscript.

## Pre-publication history

The pre-publication history for this paper can be accessed here:

http://www.biomedcentral.com/1471-2377/10/92/prepub
